# Regional occurrence, high frequency but low diversity of mitochondrial DNA haplogroup d1 suggests a recent dog-wolf hybridization in Scandinavia

**DOI:** 10.1111/j.1365-2052.2010.02069.x

**Published:** 2011-02

**Authors:** C F C Klütsch, E H Seppälä, T Fall, M Uhlén, Å Hedhammar, H Lohi, P Savolainen

**Affiliations:** * KTH-Royal Institute of Technology, Gene TechnologyRoslagstullsbacken 21, 10691 Stockholm, Sweden; † Department of Medical Genetics and Department of Basic Veterinary Sciences, University of Helsinki and Folkhälsan Institute of GeneticsHaartmaninkatu 8, P.O. Box 63, 00014 Helsinki, Finland; ‡ Department of Clinical Sciences, Swedish University of Agricultural SciencesP.O. Box 7054, 75007 Uppsala, Sweden

**Keywords:** *Canis familiaris*, domestic dog, domestication, mitochondrial DNA, spitz-type breeds

## Abstract

The domestic dog mitochondrial DNA (mtDNA)-gene pool consists of a homogenous mix of haplogroups shared among all populations worldwide, indicating that the dog originated at a single time and place. However, one small haplogroup, subclade d1, found among North Scandinavian/Finnish spitz breeds at frequencies above 30%, has a clearly separate origin. We studied the genetic and geographical diversity for this phylogenetic group to investigate where and when it originated and whether through independent domestication of wolf or dog-wolf crossbreeding. We analysed 582 bp of the mtDNA control region for 514 dogs of breeds earlier shown to harbour d1 and possibly related northern spitz breeds. Subclade d1 occurred almost exclusively among Swedish/Finnish Sami reindeer-herding spitzes and some Swedish/Norwegian hunting spitzes, at a frequency of mostly 60–100%. Genetic diversity was low, with only four haplotypes: a central, most frequent, one surrounded by two haplotypes differing by an indel and one differing by a substitution. The substitution was found in a single lineage, as a heteroplasmic mix with the central haplotype. The data indicate that subclade d1 originated in northern Scandinavia, at most 480–3000 years ago and through dog-wolf crossbreeding rather than a separate domestication event. The high frequency of d1 suggests that the dog-wolf hybrid phenotype had a selective advantage.

Studies of the global diversity for dog mitochondrial DNA (mtDNA) have shown that a homogenous gene pool is universally shared, indicating a single geographical origin for all dogs. Among 1543 dogs from across the Old World (Pang *et al.* 2009), the mtDNA haplotypes [based on 582 bp of the control region (CR)] were distributed in six phylogenetic groups, called clades A–F.

Clades A, B and C were homogenously distributed at high frequencies, normally 100%, among all populations and therefore probably formed in a single domestication event (Pang *et al.* 2009). Diversity was distinctively higher in southern East Asia than in other regions, indicating that the place of origin was East Asia (Savolainen *et al.* 2002) specifically South China or Southeast Asia (Pang *et al.* 2009). The other clades (D, E and F) had limited geographical distributions and low total frequency. Clades E and F were found exclusively in East Asia and possibly formed together with clades A, B and C. By contrast, clade D was geographically restricted to North Europe, Siberia, Southwest Asia and the Mediterranean (Angleby & Savolainen 2005; Pang *et al.* 2009), and is therefore the only mtDNA haplogroup that clearly did not originate in East Asia. Analysis of complete mtDNA genomes showed clade D to consist of two subclades (d1 and d2), which separated at least 50 000 years ago (Pang *et al.* 2009), well before the origins of dogs approximately 10 000–15 000 years ago (Clutton-Brock 1995; Pang *et al.* 2009; see also Appendix S1). The two subclades had separate geographical distributions (d1 restricted to North Eurasia and d2 to Southwest Asia and the Mediterranean) and are therefore likely have separate origins from wolf.

Subclade d2 and clades E and F were found in only a few percent of dogs within their distribution ranges. By contrast, subclade d1 had a frequency above 30% in native breeds in its core distribution area in Northern Scandinavia (Angleby & Savolainen 2005; Pang *et al.* 2009), making it the only mtDNA haplogroup that both is geographically restricted to a limited region outside East Asia and has a high frequency. Therefore, it represents the only clear sign of a major separate influx of ‘wolf genes’ into the dog gene pool.

Importantly, a haplogroup introduced through crossbreeding of wolf into an established dog population would normally, starting from a low initial frequency, remain in the minority. By contrast, haplogroups introduced through independent domestication of wolf, by a human population without dogs, would have an initial frequency of 100%. The high frequency of subclade d1 is therefore the only clear indication in the mtDNA data of a possible independent domestication of wolf, separate from that which formed clades A, B and C. Detailed knowledge about the origins of subclade d1 is, consequently, of great importance for elucidating the earliest history of the domestic dog. Therefore, we investigated the origin of mtDNA subclade d1 to find out where and when it formed and whether this was through independent domestication of wolf or through hybridization between male dog and female wolf.

We performed an extensive survey of the breeds previously known to harbour subclade d1 haplotypes [analysing 68.8% of all known female lineages, according to the pedigree data bases of the Swedish (http://kennet.skk.se/hunddata/) and Finnish (http://jalostus.kennelliitto.fi) Kennel Clubs] and of possibly related breeds and populations, based on similar morphology and/or close geographical distribution (Table S1). Hereby, we can present a comprehensive picture of the genetic and geographical diversity for mtDNA subclade d1.

We studied 582 bp of the mtDNA CR for 514 individuals (280 DNA sequences generated in this study), representing at least 328 female lineages of Scandinavian and Arctic Spitz breeds (Tables 1, S2 & S3; see Appendix S1 for exact definition of ‘lineage’).

**Table 1:** Representation of subclade d1 haplotypes among Scandinavian and Arctic spitz breeds

Breed	*n*	No. of lineages	Prop. (%) of known lineages	d1 (%)	D1 (%)	D2 (%)	D3 (%)	D4 (%)
Finnish Lapphund	23	17	85	11 (64.7)	10 (90.1)	–	1 (9.1)	–
Lapponian Herder	37	12	85.7	9 (75.0)	7 (77.8)	–	1 (11.1)	1 (11.1)
Sw. Lapphund	8	4	66.7	4 (100)	4 (100)	–	–	–
Lapphund, non-specific	4	3	–	1 (33.3)	–	–	1 (100)	–
Jämthund (Sw. Elkhound)	27	19	79.2	14 (73.7)	5 (35.7)	–	9 (64.3)	–
Norw. Elkhound (grey)	35	28	68.3	13 (46.4)	10 (76.9)	–	3 (23.1)	–
Norw. Elkhound (black)	4	3	30	3 (100)	3 (100)	–	–	–
Hälleforshund	5	5	55.5	5 (100)	4 (80)	–	1 (20)	–
Sw. Elkhound (white)	9	8	61.5	2 (25)	2 (100)	–	–	–
Finnish Spitz	15	14	87.5	1 (7.1)	–	1 (100)	–	–
Sw. Vallhund	11	7	77.8	–	–	–	–	–
Norrbottenspets	11	9	100	–	–	–	–	–
Karelian Beardog	11	11	84.6	–	–	–	–	–
Norw. Lundehund	17	15	78.9	–	–	–	–	–
Norw. Buhund	19	13	86.7	–	–	–	–	–
Icelandic Sheepdog	17	13	76.5	–	–	–	–	–
Total	**253**	**181**	**74.9**	**63**	**45**	**1**	**16**	**1**
17 Arctic spitz breeds	**261**	**147**	–	**2 (1.36)**	**2 (100)**	–	–	–

17 Arctic spitz breeds, 17 of the most common breeds and types of spitz dogs in the Arctics (e.g. Samoyed, Siberian Husky, Inuit sled dog and seven varieties of Laika; see Table S2 for a complete list); *n*, number of samples; No. of lineages, the minimum number of female lineages among the samples; Prop. (%) of known lineages, proportion (in percent) of the known female lineages in the Swedish and Finnish pedigree data bases; d1 (%), number of lineages (percent of the analysed lineages within parenthesis) having a subclade d1 haplotype; D1 (%) through D4 (%), number of lineages (percent of the lineages having a d1 haplotype within parenthesis) having haplotype D1 through D4.

Subclade d1 haplotypes were found only in breeds from Scandinavia, except for one lineage each in East Siberian and Russo-European Laika (Table 1; Table S2). A high proportion of individuals having d1 was found for some common Scandinavian breeds: Lapponian Herder (75% of investigated lineages), Jämthund (74%), Finnish Lapphund (65%), and Norwegian Elkhound (grey) (46%) and some breeds supposedly founded by few lineages had exclusively d1 haplotypes (100%). Notably, several common Scandinavian/Nordic breeds, e.g. Swedish Vallhund and Norwegian Buhund, did not have d1 haplotypes. Thus, subclade d1 was found at high frequency in all Sami-related breeds (Finnish and Swedish Lapphund, and Lapponian Herder) and in some North Scandinavian hunting dog breeds.

The high frequency of d1, above 50%, in lineages (Table 1, Fig. 1a) of a number of Scandinavian breeds, is remarkable. It is the only example of a mtDNA haplogroup found only in a specific type of dogs from a restricted geographical area and in the majority of the individuals in this area.

**Figure 1:**
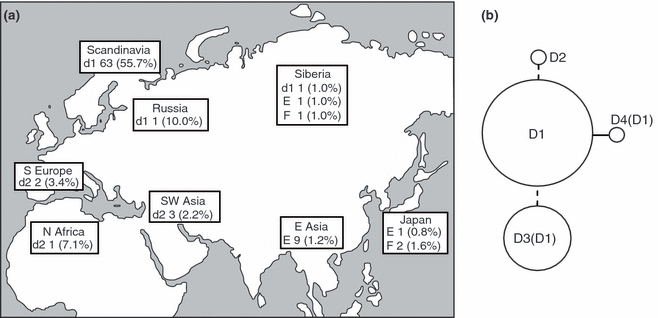
Genetic and geographical diversity for subclade d1. (a) Geographical frequency distribution of subclade d1 and the three other regionally occurring mtDNA haplogroups, d2, E and F. Number of female lineages and proportion of lineages (%) carrying the haplogroup is given for Scandinavia, Russia and Siberia; number and proportion of individuals carrying the haplogroup is given for other geographical regions, for which information about female lineages is generally lacking. (b) Minimum spanning network showing the genetic relationships between the haplotypes of subclade d1. Haplotypes (circles) are separated by a substitution (solid line) or single-base indel (broken line). ‘D3 (D1)’ and ‘D4 (D1)’ signify that D3 and D4 are not fixed haplotypes, but rather heteroplasmic mixes with D1. Circle sizes are proportional to the frequency of the haplotypes.

The fact that the d1 haplotypes were almost exclusively found among breeds from Northern Scandinavia and Finland strongly indicates that this haplotype originated in this region. Other datasets do not give further clues: Neolithic dog samples from southern Sweden (Malmström *et al.* 2008) carried only haplogroups A and C, but the samples were from outside the historical distribution of the breeds carrying d1, and among wolves no haplotypes similar to subclade d1 were found among extant populations across Eurasia, including Scandinavia (Aggarwal *et al.* 2007) or historical Scandinavian samples (Flagstad *et al.* 2003).

Despite the exhaustive sampling, which increased the number of lineages shown to carry d1 haplotypes from 20 to 63, only four previously identified haplotypes (Savolainen *et al.* 2002; see also Appendix S1) were found (Fig. 1b). The haplotypes have a star-like distribution: a central haplotype (D1, found in 45/63 (71.4%) of d1 lineages and in all breeds having subclade d1, except Finnish Spitz) surrounded by three less frequent haplotypes, two differing by a single-base indel and only one haplotype (D4), found in a single lineage, differing by a substitution. Importantly, the lineage having the D4 haplotype was heteroplasmic for the substitution separating it from D1, thus having a mixture of D1 and D4. At inspection of nine related individuals, two had non-heteroplasmic D1 (as interpreted from the Sanger chromatograms), two non-heteroplasmic D4 and five had a mixture of D1 and D4 in varying proportions. In addition, some individuals having haplotype D3 were heteroplasmic.

The central position and high frequency for haplotype D1 and the non-fixed state of D3 and D4 suggests that haplotype D1 was the founder haplotype for subclade d1, introduced from wolf to dog, and that D2–D4 have been derived by mutations in the dog population.

The low genetic diversity, with a single lineage of 63 carrying a non-fixed substitution, indicates a recent origin for subclade d1. The mean number of substitutions compared with haplotype D1 among the 63 lineages, the statistic *ρ* (Forster *et al.* 1996), is 0.016 (SEM = 0.0020) and the substitution rate for the 582 bp region has been estimated to one substitution per 40 000–155 000 years (Pang *et al.* 2009). From this, we estimate the time since the origin of dog haplogroup d1 to be 480–3000 years. Considering the non-fixed state of haplotype D4, this is most likely an overestimation (See further discussion in Appendix S1).

There is archaeological evidence for dogs in both Sweden and Siberia by 8000 years ago (Arnesson Westerdahl 1983; Lepiksaar 1984; Morey 2006). Therefore, an origin of subclade d1 less than 3000 years ago indicates that it derives from crossbreeding of wolf into an already established dog population carrying haplogroups A–C, and not from independent domestication of wolves. The high frequency of d1 haplotypes among Scandinavian dogs, therefore, warrants an alternative explanation. One possibility is that the offspring from the crossbreeding had a successful phenotype selected for by humans. If females descending from the crossbred litter were selected for during the first few generations, an increased frequency of d1 would be the result.

The sharing of the d1 haplotypes between the Lapphund breeds associated with the non-Indo-European speaking and nomadic Sami and some hunting breeds connected to Indo-European speaking farmers (Table S1) is notable. Possibly, efficient hunting and herding dogs were items of trade between the two populations. The direction of this trade is not clear, but an origin of d1 among the Sami related breeds is indicated, as all these breeds have d1 haplotypes, while only some breeds linked to the Indo-Europeans have this haplotype (Table 1).

As haplogroup d1 probably derives from dog-wolf crossbreeding, there are no clear signs in the mtDNA data that dogs were domesticated more than once (Pang *et al.* 2009). Together with haplogroups d2, E and F, haplogroup d1 represents one of only four indications of crossbreeding between female wolf and male dog through history. Whether female dog-male wolf crossbreeding has been equally rare is unclear because of the lack of comprehensive studies of paternally-inherited markers.

## Supporting information

**Appendix S1** Materials and methods, Additional text and Additional references.

**Table S1** Summary of (often anecdotal) information in the literature about breed history of Scandinavian and Arctic Spitz-type breeds.

**Table S2** Representation of subclade d1 haplotypes among Scandinavian and Arctic spitz breeds (see also Note to Table 1). In addition, the number of individuals carrying haplotypes from clades A–C, E and F is given.

**Table S3** List of samples giving mtDNA haplotype, breed, geographical origin and pedigree information (name and registration number, mother's and father's name and earliest female ancestor).

As a service to our authors and readers, this journal provides supporting information supplied by the authors. Such materials are peer-reviewed and may be re-organized for online delivery, but are not copy-edited or typeset. Technical support issues arising from supporting information (other than missing files) should be addressed to the authors.
