# Supplementary material for: Regional occurrence, high frequency but low diversity of mitochondrial DNA haplogroup d1 suggests a recent dog-wolf hybridization in Scandinavia
Source: Anim Genet. 2011 Feb;42(1):100–3. doi: 10.1111/j.1365-2052.2010.02069.x (PMC3040290; doi:10.1111/j.1365-2052.2010.02069.x)
Supplement: Supplementary file 1 [file age0042-0100-SD1.pdf]

Appendix S1. Materials and Methods, Additional text and Additional references.

Contents **Page**

Contents	Page
Materials and Methods	1
Haplotypes in subclade d1	3
New haplotypes identified in the study	3
Heteroplasmy and dating of the origin of subclade d1	3
Additional references	4

Materials and Methods

Samples

514 samples from a wide range of Spitz-type breeds from the northern parts of Eurasia and America (Table 1, Table S2, Table S3) were analyzed, to cover the potential geographical distribution of subclade d1 haplotypes. DNA sequences from 280 dogs were generated in this study, and the remaining samples were obtained from the literature (Pang *et al.* 2009).

Great care was taken to include as many as possible of the known female lineages of the breeds, according to the pedigrees in the public Swedish (http://kennet.skk.se/hunddata/) and Finnish (http://jalostus.kennelliitto.fi) Kennel Club data bases. A female lineage here means all individuals that can be traced back to a common female ancestor (founder), which consequently is the first registered individual of that lineage. It is, obviously, possible that different founders have an ancestry from a common female ancestor that lived before breed pedigree information was recorded. For individuals of known identity only samples from different female lineages were analysed. It should be noted that for the Arctic breeds, there are likely more lineages than included in these data bases, because the breeds are not indigenous

to Scandinavia. Furthermore, in order to detect potential additional diversity in the dog breeds under investigation, unregistered dogs were also collected and care was taken to obtain samples from unrelated dogs at least at the grandparent level. Sampling included also several local breeds not recognized by all Kennel Clubs, dogs with no identity information other than the breed, and dogs of no particular breed belonging to indigenous Arctic peoples (Table 1; Table S2; Table S3). For calculating the "minimum number of female lineages" for a breed (Table 1, Table S2), individuals lacking lineage information, but having a haplotype differing from those of the identified lineages of the breed, were treated as an additional lineage in the analyses, but each haplotype counted as a single lineage even if found among several individuals. The samples were collected as buccal epithelial cells using FTA-indicating cards according to the manufacturer's specifications (Whatman International, UK) or as EDTA-blood samples from which genomic DNA was extracted using a commercially available kit (Puregene, Gentra Systems, Minneapolis, MN).

DNA sequence analysis

Amplification and DNA sequencing (the sequence determined in both forward and reverse direction for all nucleotide positions) of a 582 bp long fragment of the mtDNA control region was performed as earlier described (Angleby & Savolainen 2005). Sequencing of PCR products was performed with BigDye Terminator chemistry on ABI 377 and ABI 3700 instruments (Applied Biosystems). Sequences were aligned with BioEdit (Hall 1999) and checked by eye. Comparisons of sequences and identification of haplotypes were performed with DnaSP v5 (Librado & Rozas 2009).

Calculation of time estimate

The substitution rate for the studied 582 bp region ($6.4x10^{-6}$ – $2.5x10^{-5}$ substitutions site^{-1} year^{-1}, or 1 substitution per 40,000-155,000 years) was taken from Pang *et al.* (2009), where it was calculated from the average genetic distance between dog/wolf and coyote sequences in a phylogenetic tree (a maximum likelihood evaluated neighbor-joining tree), and the time since separation of dog/wolf and coyote according to the fossil record. The rate estimate has a broad range since there is no exact calibration point for the separation time of the dog/wolf and coyote lineages; the

split may have occurred 1.5-4.5 million years ago (Nowak 2003). The time since the introduction of subclade d1 into the dog population was calculated using the ρ statistic, the mean number of substitutions for a set of sequences to their common ancestral haplotype (Forster *et al.* 1996).

Haplotypes in subclade d1

Four haplotypes (D1-D4) belonging to subclade d1 were found in the study, all of which previously described (Savolainen *et al.* 2002). A fifth haplotype (D8) earlier reported for a single individual (Pang *et al.* 2009) was upon re-sequencing found to be a D3.

New haplotypes identified in the study

Eight new haplotypes belonging to clades A, C, and E were found, one in Norrbottenspets and Finnish Spitz, one in Swedish Elkhound (white) and six in Arctic breeds. The sequences have been deposited at GenBank with accession numbers GQ896338 - GQ896345.

Heteroplasmy and dating of the origin of subclade d1

The date of origin from wolf for subclade d1 was calculated to 480-3,000 years ago based on the ρ statistic, the mean number of substitutions for a set of sequences to their common ancestral haplotype (Forster *et al.* 1996). This value was 1/63, because a single lineage out of 63 differed by a single substitution (defining the D4 haplotype) from the other 62 lineages, which were all identical ignoring indel variation. However, the calculation of ρ was based on treating the single substitution as fixed, while it in reality occurred as a heteroplasmic mix with the wild-type. The estimated age of subclade d1 is therefore likely an overestimation.

Furthermore, the heteroplasmic state of haplotype D4 (and also D3 which was heteroplasmic for the indel distinguishing it from D1) represents in itself an indication of a young age of the subclade, since it does not seem likely that the heteroplasmic state would have been transferred through the generations during thousands of years. For a neutral mitochondrial heteroplasmic variant, the time to fixation has been estimated to approximately 200 generations in humans and chinook salmon (White *et al.* 2008). Thus, assuming a generation time of 3-5 years in dogs (Fuller *et al.* 2003) this would indicate an origin less than 1,000 years ago for the D3 and D4 mutations.

To conclude, regardless whether dogs originated approximately 10,000-15,000 years ago (Clutton-Brock 1995; Pang *et al.* 2009), or as early as 31,700 years ago as suggested by Germonpré *et al.* (based on archaeological remains of canids with morphometrics falling between those of wolf and dog), subclade d1 obviously originated at a much later date, at which time dog populations had been established globally, including Scandinavia (Arnesson Westerdahl 1983; Morey 2006). Therefore, an origin of subclade d1 from a dog-wolf crossbreeding seems firmly established.

Additional references

American Kennel Club (2006) *The complete dog book.* 20th edition. Ballantine Books, New York.

Fuller K.F., Mech L.D. & Cochrane J.F. (2003) Wolf population dynamics. In: *Wolves: behavior, ecology, and conservation*. (Ed. by L.D. Mech & L. Boitani), pp. 161–91. University of Chicago Press, Chicago.

Germonpré M., Sablin M.V., Stevens R.E., Hedges R.E.M., Hofreiter M., Stiller M., Després V.R. (2009) Fossil dogs and wolves from Paleolithic sites in Belgium, the Ukraine and Russia: Osteometry, ancient DNA and stable isotopes. *Journal of Archaeological Science* **36**, 473–90.

Hall T.A. (1999) BioEdit: a user-friendly biological sequence alignment editor and analysis program for Windows 95/98/NT. *Nucleic Acids Symposium Series* **41**, 95-8.

Jahnsson B.O. & Samuelsson R.B. (1996) *Nordliga Jaktspetsar*. ICA Bokförlag AB Västerås (in Swedish).

Librado P. & Rozas J. (2009) DnaSP v5: A software for comprehensive analysis of DNA polymorphism data. *Bioinformatics* **25,** 1451-2.

Lindholm Å. (2008) Svenska Hundraser-ett kulturarv. Sellin & Partner Bok och Idé AB, Stockholm.175 pp. (in Swedish & English).

MacRury I.K. (1991) *The Inuit Dog: Its Provenance, Environment and History*. Master thesis, University of Cambridge.

Nowak R.M. (2003) Wolf evolution and taxonomy. In: *Wolves: Behavior, ecology, and conservation* (Ed. by L.D. Mech & L. Boitani), pp. 239-58. University of Chicago Press, Chicago.

Oliehoek P.A. (1999) *Inbreeding, effective population size, mean kinship and cluster analysis in the Icelandic Sheepdog as a small Population*, Student thesis Wageningen, Mimeo 56.

Parker H.G., Kim L.V., Sutter N.B., Carlson S., Lorentzen T.D., Malek T.B., Johnson G. S., DeFrance H.B., Ostrander E.A. & Kruglyak L. (2004) Genetic structure of the purebred domestic dog, *Science* **304**, 1160–66.

White D.J., Wolff J.N., Pierson M. & Gemmell N.J. (2008) Revealing the hidden complexities of mtDNA inheritance. *Molecular Ecology* **17**, 4925-42

Willes R. (2003) *All världens hundraser*. MB Förlag, Bromma (in Swedish).
